# Supplementary material for: Acacia senegal Budmunchiamines as a Potential Adjuvant for Rejuvenating Phenicol Activities towards Escherichia coli-Resistant Strains
Source: Int J Mol Sci. 2023 May 15;24(10):8790. doi: 10.3390/ijms24108790 (PMC10218413; doi:10.3390/ijms24108790)
Supplement: Supplementary file 1 [file ijms-24-08790-s001.zip › ijms-2373162-supplementary.pdf]

Supplementary Data

Table S1: Bacterial strains used in this study

**Strains**	**Description**	**Reference**
E. coli		
AG100	Parental *E. coli* K-12 Porin $^{+}$; basal efflux	[1]
AG100A	AG100 acrAB: Kan^{r} non (AcrAB^{-}); porin $^{+}$	
AG102	AG100 overexpressing the AcrAB pump, porin^{+}	
K. aerogenes		
Ka 289	KAN-sensitive derivative of EA27, Porin^{-}	[2]
Ka 298	EA289 TolC: Kan^{r}; porin^{-}	[2]
Ka ATCC15038	Parental ATCC strain : porin^{+}; normal efflux	[3]
Ka CM 64	CHL^{r} variant obtained from ATCC 13048 overexpressing the AcrAB pump; porin^{+}	[4]

1. Vergalli, J.; Dumont, E.; Cinquin, B.; Maigre, L.; Pajovic, J.; Bacqué, E.; Mourez, M.; Réfrégiers, M.; Pagès, J.-M. Fluoroquinolone Structure and Translocation Flux across Bacterial Membrane. *Scientific Reports* **2017**, *7*, 9821.
2. Masi, M.; Pagès, J.-M.; Pradel, E. Overexpression and Purification of the Three Components of the Enterobacter Aerogenes AcrA–AcrB–TolC Multidrug Efflux Pump. *Journal of Chromatography B* **2003**, *786*, 197–205.
3. Mallea, M.; Chevalier, J.; Bornet, C.; Eyraud, A.; Davin-Regli, A.; Bollet, C.; Pages, J.-M. Porin Alteration and Active Efflux: Two in Vivo Drug Resistance Strategies Used by Enterobacter Aerogenes. *Microbiology* **1998**, *144*, 3003–3009.
4. Ghisalberti, D.; Masi, M.; Pagès, J.-M.; Chevalier, J. Chloramphenicol and Expression of Multidrug Efflux Pump in Enterobacter Aerogenes. *Biochemical and biophysical research communications* **2005**, *328*, 1113–1118.

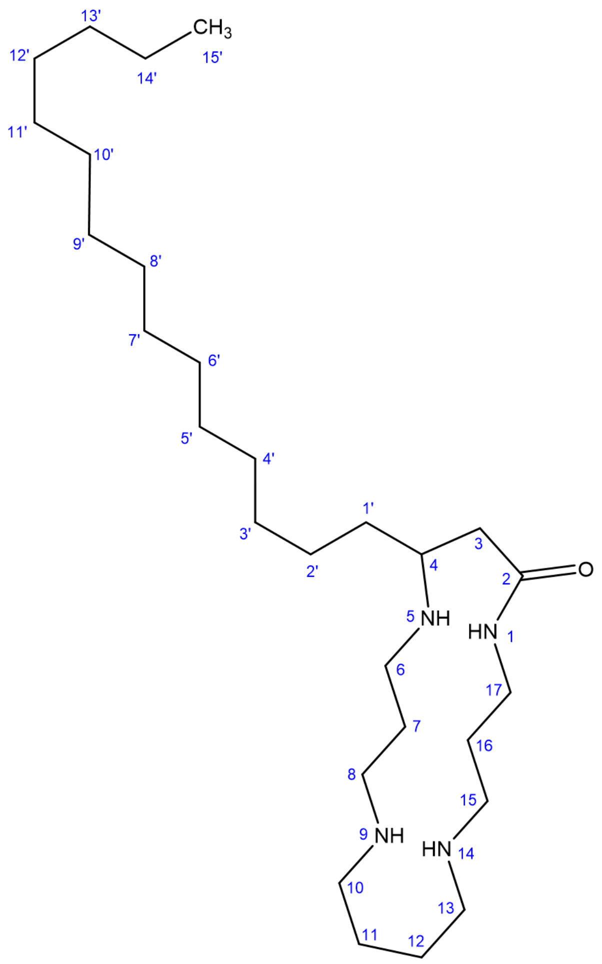

Figure S1: Numbering of the Budmunchiamine L5 atoms.

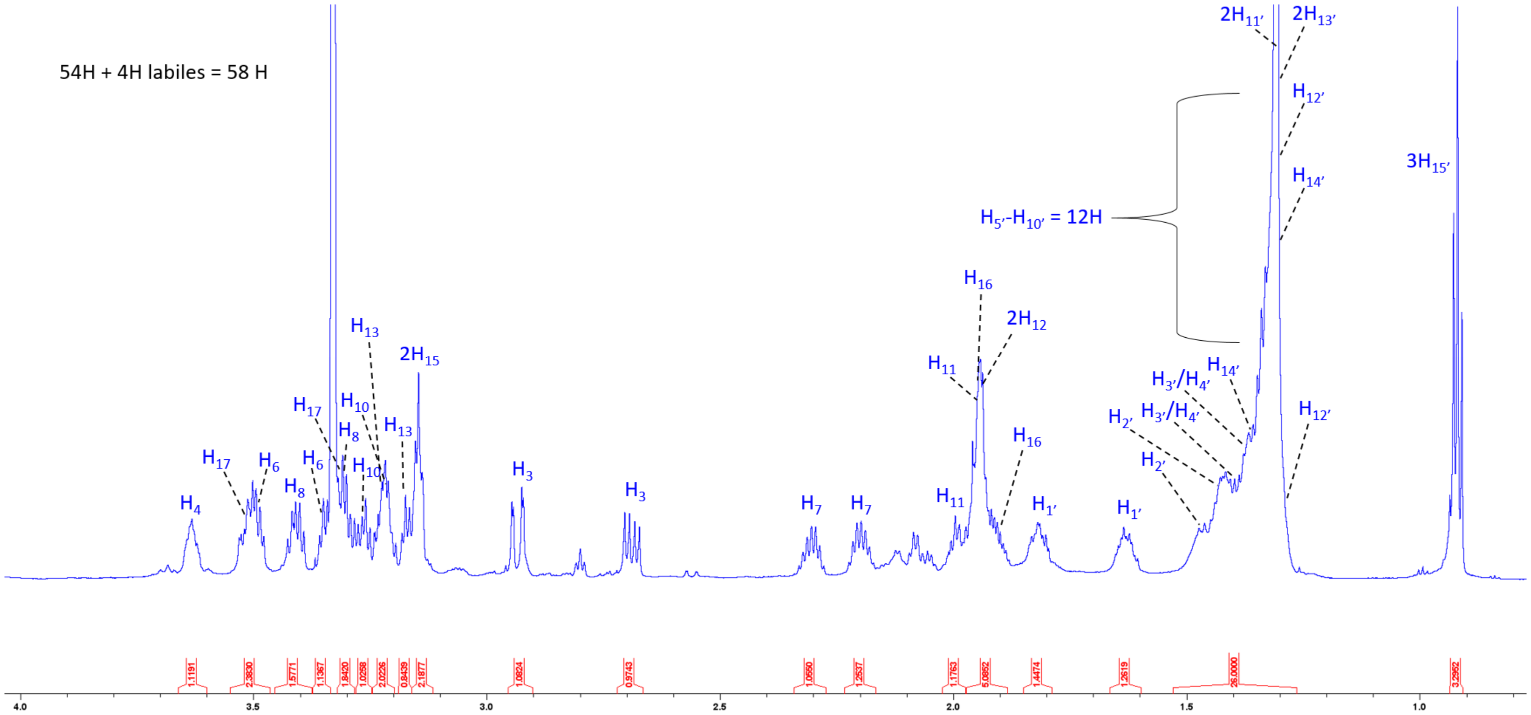

Figure S2: ^{1}H spectrum of Budmunchiamine L5.

International Journal of *Molecular Sciences*

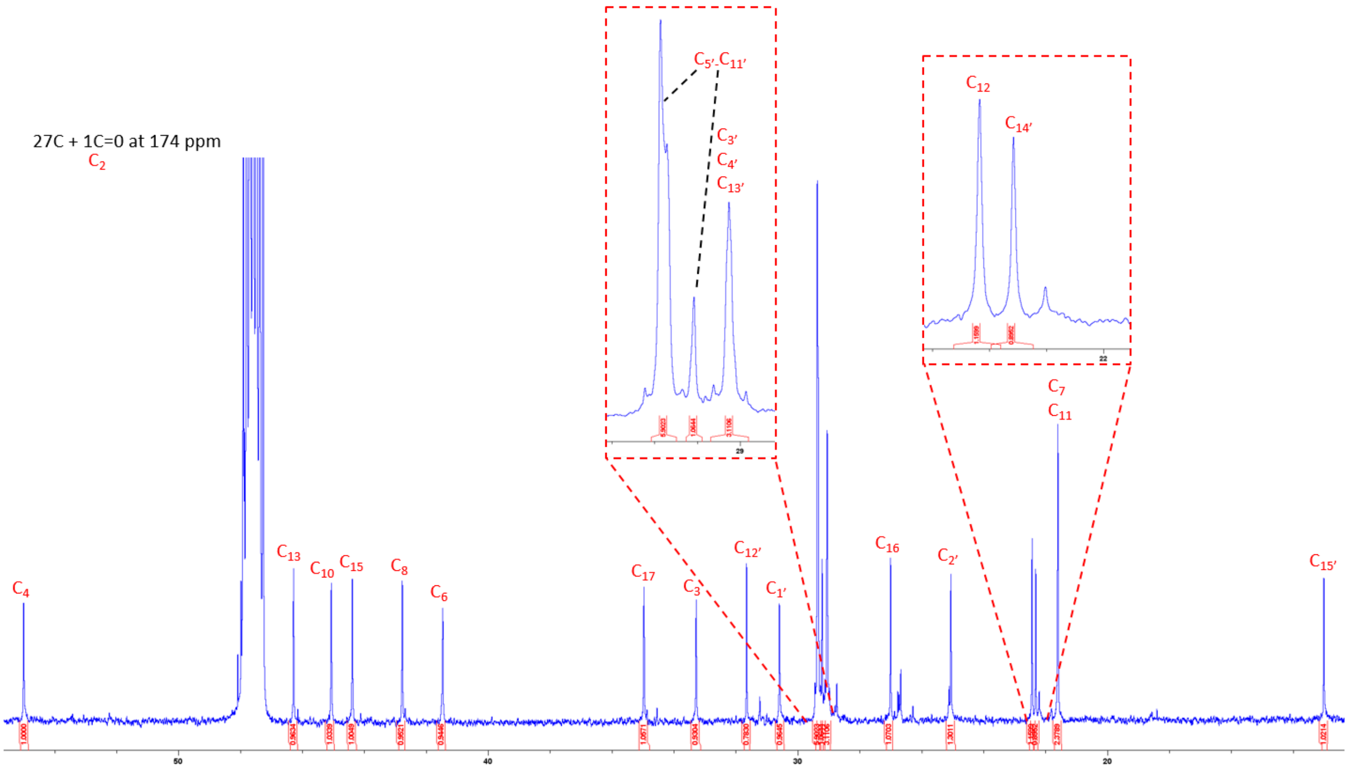

Figure S3: ^{13}C spectrum of Budmunchiamine L5.

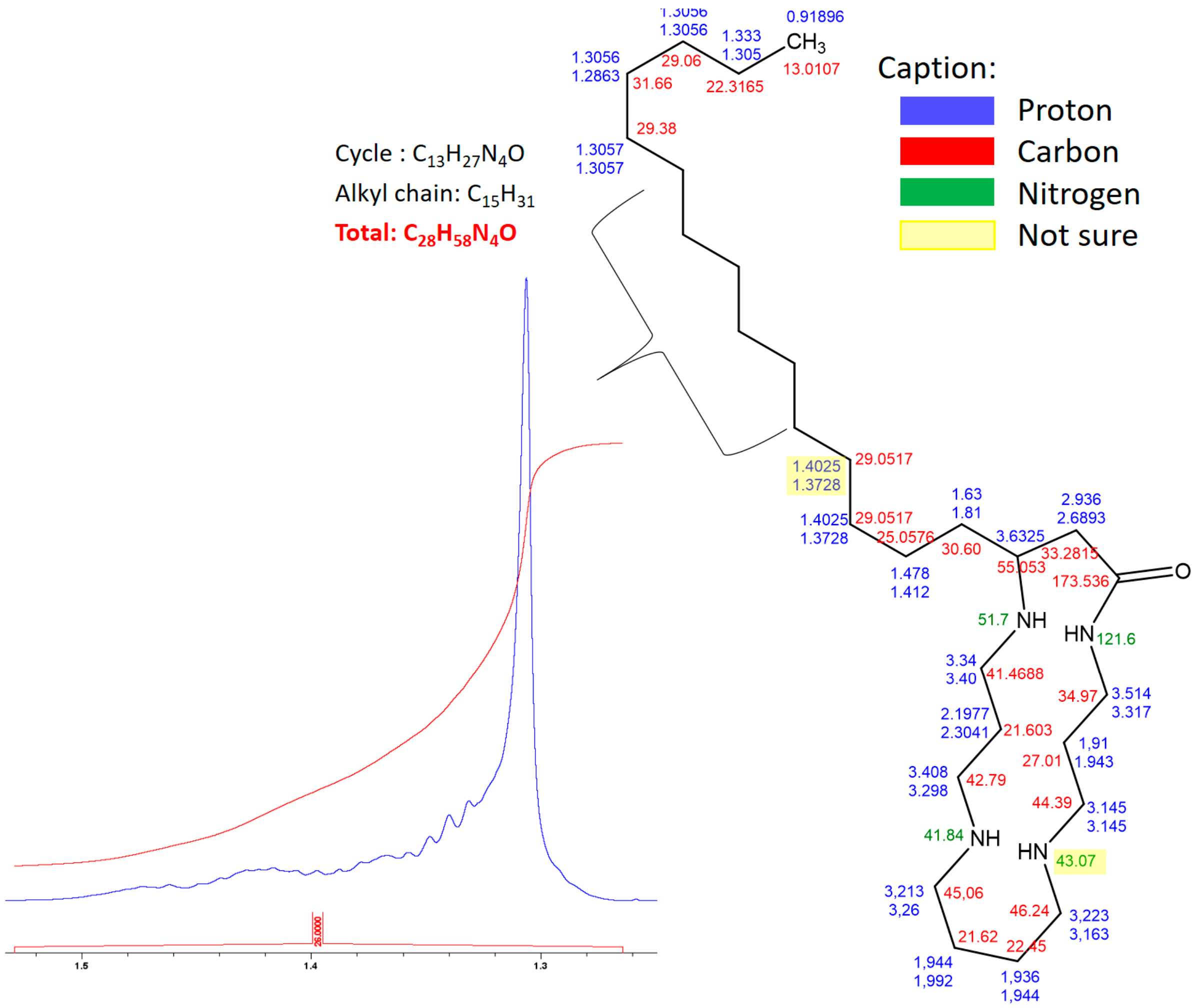

Figure S4: Chemical shift assignment of Budmunchiamine L5 $^{1}H/^{13}C$ and ^{15}N.

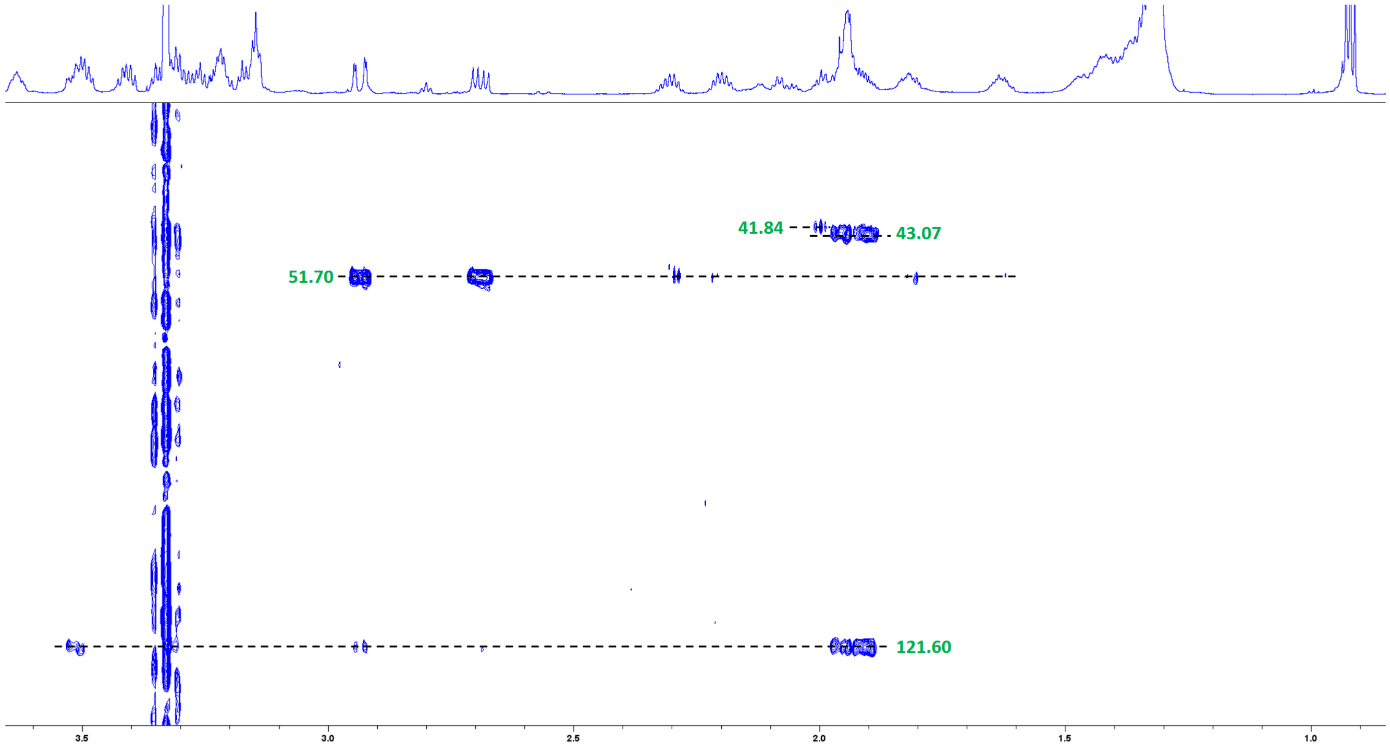

Figure S5: Chemical shift assignment of Budmunchiamine L5 $^{1}H/^{15}N$.
